# Healthcare Professionals’ Experiences During the COVID-19 Pandemic in Sudan: A Cross-Sectional Survey Assessing Quality of Life, Mental Health, and Work-Life-Balance

**DOI:** 10.3389/ijph.2023.1605991

**Published:** 2023-08-30

**Authors:** Mohamed Salih Mahfouz, Sara Amir Osman, Badreldin Abdelrhman Mohamed, Esra Ali Mahjoub Saeed, Mohajer Ibrahim Hassan Ismaeil, Rafiaa Ali Abdalla Elkhider, Merdi Ahmed Orsud

**Affiliations:** ^1^ Department of Family and Community Medicine, Faculty of Medicine, Jazan University, Jazan, Saudi Arabia; ^2^ Faculty of Medicine and Health Sciences, Omdurman Islamic University, Khartoum, Sudan; ^3^ College of Applied Medical Sciences, King Saud University, Riyadh, Saudi Arabia; ^4^ Faculty of Medicine, University of Khartoum, Khartoum, Sudan; ^5^ Faculty of Medicine, Alzaiem Alazhari University, Khartoum, Sudan; ^6^ Independent Researcher, Jazan, Saudi Arabia; ^7^ Mathematics Department, College of Science, Jazan University, Jazan, Saudi Arabia

**Keywords:** HRQOL, general anxiety disorder, healthcare professions, work life balance, Sudan

## Abstract

**Objectives:** The main objective of this research was to investigate the Work-life balance (WLB), mental health, and quality of life and their associated factors among Healthcare Professionals (HCPs) in Sudan during the peak of the COVID-19 pandemic during 2021.

**Methods:** An observational cross-sectional web-based survey was conducted during August-December 2021 among a sample of 430 HCPs working in the hospitals of four Sudanese states. The study used the WHOQoLBREF scale, Work-Life Balance Scale, and General Anxiety Disorder (GAD-7).

**Results:** HCPs reporting a poor quality of life made up 33.5% (95% CI 29.1–38.0), while those reporting worse WLB made up 52.6% (95% CI 47.8–57.2). HCPs reporting moderate to severe anxiety symptoms accounted for 35.8% (95% CI 31.4–40.5). The Multiple Regression model indicated that an increase in the anxiety scores is associated with a decrease in Health-related Quality of Life (HRQoL) (*β*= 0.831, *p* < 0.05). Female HCPs exhibited 4.53-fold lower HRQoL scores than their male colleagues (*p* < 0.05).

**Conclusion:** Approximately one-third of the HCPs in Sudan reported low HRQoL and suffered from moderate to severe anxiety, while a large portion of them had an unequal work-life balance during COVID-19. Health policies addressing these factors are needed to improve the quality of health of HCPs.

## Introduction

The timeline of COVID-19 indicated that the first case was identified in China in December 2019 before spreading to every corner of the Globe, causing the most unprecedented public health crisis in recent decades [1]. In Sudan, the first case was reported on the 13th of March 2020, and by May 2021 the infection curve showed approximately 33,104 confirmed cases, 2,349 deaths, and 26,795 recovered cases [2].

The COVID-19 pandemic has a significant burden on all aspects of life, not limited to population health. Its impact extended to economic slowdown, Gross Domestic Product (GDP) growth and people’s jobs and livelihoods. As a developing country, Sudan’s healthcare system is already flooded by structural problems and this situation has been aggravated by COVID-19 [3]. Sudan’s healthcare system is characterized by limited resources, and the available information showed that the overall government health expenditure is very low and the health sector is under-funded [4].

Healthcare Professions (HCPs) were at the heart of the storm of the COVID-19 pandemic, facing multiple challenges in treating patients with COVID-19: reducing the spread of infection and developing prevention strategies. Healthcare workers were more likely to contract SARS-CoV-2 compared to the general population. In addition, during the COVID-19 pandemic, in some countries, female healthcare workers reported higher rates of mental health problems and burnout compared to male healthcare workers [5]. Furthermore, female health workers have double the rate of COVID-19 infections compared to their male colleagues [6].

Work-life balance (WLB) is defined as a high level of role engagement in professional activities and personal commitments with minimal conflict between social roles in work and non-work-life. Normally, the term encompasses three dimensions: time balance, involvement balance, and satisfaction balance [7]. The concept also includes burnout prevention and stress management as the level of productivity is increasingly associated with time to relax and recharge [8]. Stress at work is the psychological and physical state that results when a person’s resources cannot adapt or cope with the pressures of the situation [9]. Poor work settings can be accompanied by unhealthy consequences, including marital conflicts, shortened life expectancy, and immune system impairment [10, 11]. Quality of life (QoL) is the person’s perception of his/her position in life in the context of the culture and value systems [12].

According to the World Health Organization (WHO), the perceived QoL is not only the absence of physical disease but extends to the evaluation of an individual’s psychological, social, and environmental conditions [13]. QoL is measured using eleven indicators: education, wealth, the environment, employment, physical and mental health, safety, recreation and rest time, social belonging, religious beliefs, security, and freedom [13]. Improving QoL reflects in the employees, organization, and consumers and leads to improving the quality of care, strengthening organizational commitment, and increasing productivity [13].

Recent studies on QoL documented the strong association between QoL and the mental health of HCPs during the COVID-19 pandemic [14–17]. HCPs are dealing with a large number of confirmed or suspected cases under massive psychological and physical stress [18–20]. Increasingly published reports postulated that mental health among HCPs was affected and the prevalence of depression and anxiety among HCPs during the COVID-19 pandemic was (37%) and (40%), respectively [21]. A study conducted in 34 Chinese hospitals measured the psychological reactions of healthcare workers and found high rates of sadness (50%), anxiety (42%), insomnia (34%), and distress (72%) [22]. Similarly, rates of depression, anxiety, and post-traumatic stress disorder (PTSD) in healthcare workers ranged from (8.90% to (50.45) %, (10.4%) to (44.6%), and (32%) to (71.5%), respectively, in a recent systematic review [23]. On the other hand, work-life balance is closely correlated with burnout and mental health [19, 20, 24]. Burnout of HCPs and reduction in quality of life is related to an increased number of medical errors [25]. Some studies have found that work-life balance is linked to negative outcomes such as anxiety, depression, and mental health issues [26, 27].

There is a significant amount of literature available on COVID-19 and its impact on Quality of Life, Mental Health, and Work-Life Balance. However, there are still some gaps in the research regarding the relationship between these three factors during COVID-19 among HCPs. Further, there is a scarcity of published reports on work-life balance, stress, and quality of life among healthcare workers in Sudan during the COVID-19 pandemic. Hence, the main objective of this research was to investigate the work-life balance, mental health, and quality of life and their associated factors among HCPs in Sudan.

## Methods

### Study Design, Population, and Place

An observational cross-sectional survey targeted the HCPs in Sudan hospitals. The target population includes all HCPs of different specialties and departments in the four selected States. Work settings included intensive care units, inpatient units, outpatient clinics, pharmacies, clinical labs, radiology, and nursing Departments. The main inclusion criteria were HCPs who were actively working in hospitals with COVID-19 wards, and working for at least three months in the study setting.

The Republic of Sudan comprises 18 states, is populated by 45.70 million people as of the 2022 population estimate, and occupies 1,886,068 square kilometers. In our study, we selected four states representing the whole country, namely, North Kordofan, Al-Qadarif, El Gazira, and Khartoum, for this research. These four states are situated in western, eastern, and central Sudan, with Khartoum serving as the capital. These states are geographically diverse and provide a fairly accurate representation of the country as a whole.

### Sampling Procedures

For the purpose of this study, a sample of 440 HCPs were estimated. This number was determined using the standard formula for cross-sectional surveys (Lwanga, and Lemeshow, 1991) [28], which is the initial sample size n = [(z^2^ *p * 1-p)]/d^2^. The terms of the equation are as follows: n is the required sample size; p is the attribute studied; Z is the standardized variable that corresponds to a 95% level of confidence and d is the desired marginal error.

Since we are interested in three domains (Work life balance, mental health, and QoL) a value of (0.5) for p is reasonable because it gives the maximum possible sample size. Assigning 0.05 for the marginal error and z = 1.96, the initial sample size was 400 participants and accounting for 20% non-repose rate the final size was 480 HCPs.

The sampling design was three-stage cluster random sampling; each Sudanese state was regarded as an independent cluster. Stage one involved a random selection of four states out of 16 of the whole of Sudan. In stage two, we selected one governmental hospital and another private one. We used probability proportional to size sampling (PPS) to determine the number of participants in the selected health institution. In the last step, we used a snowball technique to select healthcare professionals (HCPs) from each institution. The questionnaire link was then distributed to these HCPs through social networks like Facebook, Instagram, LinkedIn, and WhatsApp in their respective hospitals.

### Data Collection and Study Instruments

A web-based questionnaire was used for data collection. The data collection tool consists of four parts. The first is sociodemographic information (age, gender, marital status, occupation, job period, chronic disease, and Tobacco use). The second part is the Work-life Balance scale (eight items) for measuring work-life climate. The work-life climate scale asks, “During the past week, how often did this occur?” followed by phrases such as: skipped a meal, arrived home late from work, or had difficulty sleeping. The response scale for the work-life climate items ranges from: rarely or none of the time (less than 1 day); some or a little of the time (1–2 days); occasionally or a moderate amount of time (3–4 days); all of the time (5–7 days); and not applicable. Work settings with less frequent work-life climate difficulties (lower scores) have HCPs with healthier work-life-balance [20].

The third section is measured by the General Anxiety Disorder (GAD-7) questionnaire, a validated seven-item assessment (Spitzer, et al, 2006) [29]. The tool is designed to assess the patient’s health status during the previous 14 days. The items require four responses: not difficult at all, somewhat difficult, very difficult and extremely difficult. The total scale scores for the seven items range from 0 to 21 and are categorized as follows; 0–4: minimal anxiety; 5–9: mild anxiety; 10–14: moderate anxiety and 15–21: severe anxiety.

The final part of the instrument is the World Health Organization Quality of Life-BREF (WHQoL-BREF). It is a self-administered 26-item instrument categorized into four domains (physical, psychological, social, and environmental). Each instrument item is scored from 1 to 5 on a response scale. Higher total scores indicate higher QoL [13]. The instruments are available for scientific purposes without commercial use. A pilot study was conducted among 30 HCPs working in Khartoum Hospitals to test the questionnaire applicability and understanding before starting the actual research. The data from the pilot study were not included in the main study. The internal consistency was calculated and evaluated for the different measures and produced a satisfactory result. The final results of the internal consistency based on Cronbach’s alpha are presented in the results section.

### Data Management and Statistical Analysis

The IBM SPSS Statistics for Windows, Version 25.0. Armonk, NY: IBM Corp software program was used for data analysis. Descriptive statistics based on simple tabulations, frequencies, and percentages were used. The normality of continuous variables was assessed using the Shapiro-Wilk test. Means with their standard deviation were calculated and used to describe the total scores of the four domains of QoL, the scores of WLB and GAD-7. Categorical variables were described as frequency and percentage. To assess the differences in the demographic, workplace, QoL, and Anxiety characteristics, we compared the means of continuous variables using the Student’s t-test and One Way ANOVA respectively. The differences in percentages of categorical variables between the two groups was assessed using the Chi-squared test/Fisher exact test. A Multiple Linear regression model was used to assess predictors of the overall score of quality of life among the healthcare Professions. The regression model analyzed the relationship between HRQoL (the dependent variable) and several independent variables including total Anxiety scores, WLB scores, tobacco use, history of workplace violence, and demographics such as age, gender, marital status, and profession. The regression model was assessed for Multicollinearity and other assumptions underlying OLS technique. The model was also assessed for Goodness of fit using an F test and the coefficient of multiple determination (R^2^.). A *p*. value of less than 0.05 was considered significant.

### Ethical Consideration

This study was conducted according to the ethical standards of Sudan and the Helsinki declaration. The IRB committee of Al-Gadarif University approved the study protocol ref GU/FM/REC/Q3.7.12.4 dated July 2021. All the participants read, understood, and signed the study consent. The data was stored under a high confidentiality level without names to protect participants’ privacy.

## Results

Of the 480 anticipated responses, a total of 430 HCPs were included in the analysis providing a response rate of 89.6%. The background characteristics of the HCPs are presented in Table1. Most participants were female (*N* = 276, 64.2%), single HCPs (*N* = 365, 84.9%), in the age group (25–34) years (*N* = 285, 66.3%) The majority of healthcare professionals were Physicians (*N* = 293, 68.1%). Most of them had less than 4 years of experience (N = 330, 76.7%) and were working Morning shifts (*N* = 249, 57.9%). One-fifth of HCPs reported verbal violence during the COVID-19 pandemic (*N* = 98, 22.8%) and only (*N* = 47, 10.9%) were Tobacco users.

**TABLE 1:** Participants’ demographic and professional characteristics, (Sudan, 2021).

Characteristic (*N* = 430)		Count	%
Gender	Male	154	35.8
	Female	276	64.2
Marital status	Single	365	84.9
	Married	65	15.1
Age groups (years)	18–24	123	28.6
	25–34	285	66.3
	35–50	22	5.1
Profession	Physician	293	68.1
	Nurse	37	8.6
	Pharmacist	28	6.5
	Technician	22	5.1
	Other profession	50	11.6
Years of Experience	Less than 4	330	76.7
	4–9	84	19.5
	10–20	16	3.7
Working Shifts	Morning	249	57.9
	Evening	84	19.5
	Both	97	22.6
Type of violence during COVID-19	Verbal	98	22.8
	Physical	6	1.4
	Intimidation	16	3.7
	None	310	72.1
Tobacco use	Yes	47	10.9
	No	383	89.1

Table 2 summarizes the mean scores for the four domains of WHOQoL, WLB, and GAD-7 according to HCPs’ background characteristics**.** The WLB mean for the HCPs was [17 ± 5.3, 95% CI = 16.6–17.6], compared with [8 ± 5.2, 95% CI = 7.1–8.2] for the GAD-7 total score. The same Table showed a significant difference (*p* < 0.05) in mean scores for social relationship QoL domain according to the variable years of experiences. Moreover, there was a significant difference (*p* < 0.05) in mean scores according to age groups in the environment domain, GAD-7, and WLB. The type of violence mean scores also differ significantly (*p* < 0.05) across the four WHOQol domains, WLB and GAD-7. The mean scores according to Tobacco use were also significantly different for WLB and Physical QoL domain (*p* < 0.05).

**TABLE 2:** The Mean and SD scores of the health-related Quality of Life Domains, Work-life balance, and General Anxiety Disorder according to healthcare professionals background characteristics, (Sudan, 2021).

Characteristic		WHOQoL Domains^a^								WLB^b^		GAD^b^	
		Physical		Psychological		Social relationships		Environment					
		Mean	SD	Mean	SD	Mean	SD	Mean	SD	Mean	SD	Mean	SD
Gender	Male	56	(19.3)	58	(20.2)	49	(24.3)	42	(18.1)	17	(5.4)	8	(6.0)
	Female	52	(19.6)	55	(18.8)	47	(22.5)	41	(16.6)	17	(5.2)	8	(5.4)
Marital status	Single	54	(19.0)	56	(19.5)	44^c^	(21.7)	41	(16.7)	17^c^	(5.2)	8	(5.7)
	Married	51	(22.4)	56	(19.1)	66	(22.5)	42	(19.4)	19	(5.7)	8	(5.6)
Age groups (years)	18–24	57	(19.4)	59	(19.9)	48	(23.0)	44^d^	(16.7)	19^d^	(5.4)	7^d^	(5.4)
	25–34	52	(19.5)	55	(19.0)	47	(23.3)	40	(17.1)	17	(5.2)	8	(5.7)
	35–50	52	(20.1)	56	(20.3)	51	(22.8)	41	(18.4)	16	(4.6)	8	(5.2)
Profession	Physician	52^d^	(19.2)	55^d^	(19.6)	47	(23.0)	40	(17.8)	16^d^	(5.1)	8	(5.7)
	Nurse	63	(18.6)	68	(18.9)	53	(22.2)	46	(18.7)	19	(5.9)	6	(4.4)
	Pharmacist	51	(18.3)	57	(18.3)	52	(25.5)	40	(14.2)	20	(4.8)	7	(3.7)
	Technician	57	(19.9)	58	(13.8)	49	(23.8)	43	(12.2)	19	(4.6)	7	(5.8)
	Other profession	52	(21.3)	55	(18.9)	45	(22.8)	44	(14.8)	19	(5.2)	8	(6.7)
Years of Experience	Less than 4	54	(18.9)	57	(18.6)	47^d^	(22.3)	41	(16.3)	17	(5.4)	8	(5.6)
	4–9	51	(21.4)	55	(22.1)	52	(27.0)	43	(19.8)	17	(5.2)	8	(5.9)
	10–20	51	(22.4)	53	(20.6)	42	(16.1)	39	(18.4)	16	(4.6)	9	(6.0)
Working Shifts	Morning	54	(19.4)	57	(18.9)	49	(23.4)	43	(17.2)	18^d^	(5.2)	8	(5.6)
	Evening	52	(20.1)	55	(19.1)	46	(23.9)	40	(15.5)	18	(5.0)	7	(6.0)
	Both	53	(19.6)	56	(20.8)	44	(21.4)	38	(17.9)	14	(5.0)	8	(5.4)
	No	56	(19.6)	58	(18.8)	50	(22.9)	44	(16.8)	18	(5.3)	7	(5.5)
Type of violence during COVID-19	Verbal	48^d^	(19.2)	51^d^	(19.8)	41^d^	(23.0)	35^d^	(17.5)	15^d^	(4.7)	9^d^	(5.9)
	Physical	59	(8.7)	57	(9.7)	43	(20.7)	40	(12.4)	15	(4.5)	7	(2.2)
	Intimidation	44	(14.3)	47	(21.2)	41	(21.7)	35	(12.3)	14	(3.9)	12	(5.8)
	None	56	(19.6)	58	(18.9)	50	(22.9)	44	(16.8)	18	(5.3)	7	(5.5)
Tobacco use	Yes	47^c^	(19.2)	53	(20.1)	46	(24.1)	37	(20.0)	15^c^	(5.0)	7	(6.0)
	No	54	(19.5)	57	(19.3)	48	(23.0)	42	(16.7)	17	(5.3)	8	(5.6)
Healthcare professionals		53	19.6	56	(19.4)	48	(23.1)	41	(17.1)	17	(5.3)	8	(5.2)
95% CI		51.5–55.2		54.4–58.0		45.4–49.7		39.5–42.8		16.6–17.6		7.1–8.2	
Cronbach’s alpha		0.812		0.739		0.400		0.744		0.826		0.899	

Abbreviations: HCP, Healthcare professionals; GAD, General Anxiety Disorder; WLB, work Life Balance; SD, Standard Deviation.

The max value for HRQoL Domains, WLB and GAD, were 100, 32 and 16 respectively.

^a^ Higher score is favorable.

^b^ Lower score is favorable.

^c^ Significant at the 0.05 level, based on the independent sample *t*-test.

^d^ Significant at the 0.05 level, based on One-way ANOVA test.

Marital status, profession, or occupation, working shifts, workplace violence, type of violence and Tobacco use, were positively associated worse WLB (*p* < 0.05 for all). Further, experiencing workplace Violence, and certain types of violence were also positively associated with moderate to severe anxiety among the study participants (*p* < 0.05 for all). There were more female than male respondents with poor quality of life as well as more pharmacists than other occupations reporting workplace violence, but without documenting a statistically significant association (*p* > 0.05 for all) (Table 3). As shown in the same Table, the proportion of HCPs with poor HRQoL was 33.5% (95% CI 29.1–38.0), while the percentage of those reporting worse WLB was 52.6% (95% CI 47.8–57.2). HCPs reporting symptoms of moderate to severe anxiety accounted for 35.8% (95% CI 31.4–40.5) of the study participants.

**TABLE 3:** Factors associated with poor Quality of life, worse Work-life balance, and moderate to severe Anxiety among healthcare professionals, (Sudan, 2021).

Characteristic		Poor QoL		*p*-value	Worse WLB		*p*-value	Moderate to severe anxiety		*p*-value
		No	%		No	%		No	%	
Gender	Male	48	(31.2)	0.580	76	(49.4)	0.990	54	(35.1)	0.059
	Female	96	(34.8)		150	(54.3)		100	(36.2)	
Marital status	Single	123	(33.7)	0.827	181	(49.6)	0.003	126	(34.5)	0.185
	Married	21	(32.3)		45	(69.2)		28	(43.1)	
Age groups (years)	18–24	33	(26.8)	0.160	73	(59.3)	0.186	34	(27.6)	0.081
	25–34	102	(35.8)		143	(50.2)		111	(38.9)	
	35–50	9	(40.9)		10	(45.5)		9	(40.9)	
Profession	Physician	100	(34.1)	0.477	134	(45.7)	0.001	112	(38.2)	0.251
	Nurse	10	(27.0)		22	(59.5)		9	(24.3)	
	Pharmacist	13	(46.4)		20	(71.4)		7	(25.0)	
	Technician	6	(27.3)		15	(68.2)		6	(27.3)	
	Other profession	15	(30.0)		35	(70.0)		20	(40.0)	
Years of Experience	Less than 4	111	(33.6)	0.911	173	(52.4)	0.718	109	(33.0)	0.082
	4–9	27	(32.1)		46	(54.8)		37	(44.0)	
	10–20	6	(37.5)		7	(43.8)		8	(50.0)	
Working Shifts	Morning	82	(32.9)	0.654	151	(60.6)	<0.001	93	(37.3)	0.928
	Evening	26	(31.0)		48	(57.1)		28	(33.3)	
	Both	36	(37.1)		27	(27.8)		33	(34.0)	
Work place violence	Yes	48	(40.0)	0.075	42	(35.0)	<0.001	55	(45.8)	0.007
	No	96	(31.0)		184	(59.4)		99	(31.9)	
Type of violence during COVID-19*	Verbal	40	(40.8)	0.271	33	(33.7)	<0.001	42	(42.9)	0.006
	Physical	1	(16.7)		2	(33.3)		1	(16.7)	
	Intimidation	6	(37.5)		6	(37.5)		11	(68.8)	
	None	97	(31.3)		185	(59.7)		100	(32.3)	
Tobacco use	Yes	21	(44.7)	0.085	14	(29.8)	0.001	14	(29.8)	0.361
	No	123	(32.1)		212	(55.4)		140	(36.6)	
All HCPs		144	(33.5)		226	(52.6)		154	(35.8)	
95% CI		29.1–38.0			47.8–57.2			31.4–40.5		

Abbreviations: HCPs, Healthcare professionals; WLB, work Life Balance.

The cutoff for Moderate to severe anxiety symptoms was >8, poor QoL <50 and worse WLB >16.

*p*-value was based on Chi squared test; *p-value is based on Fisher Exact test.

**FIGURE 1:**
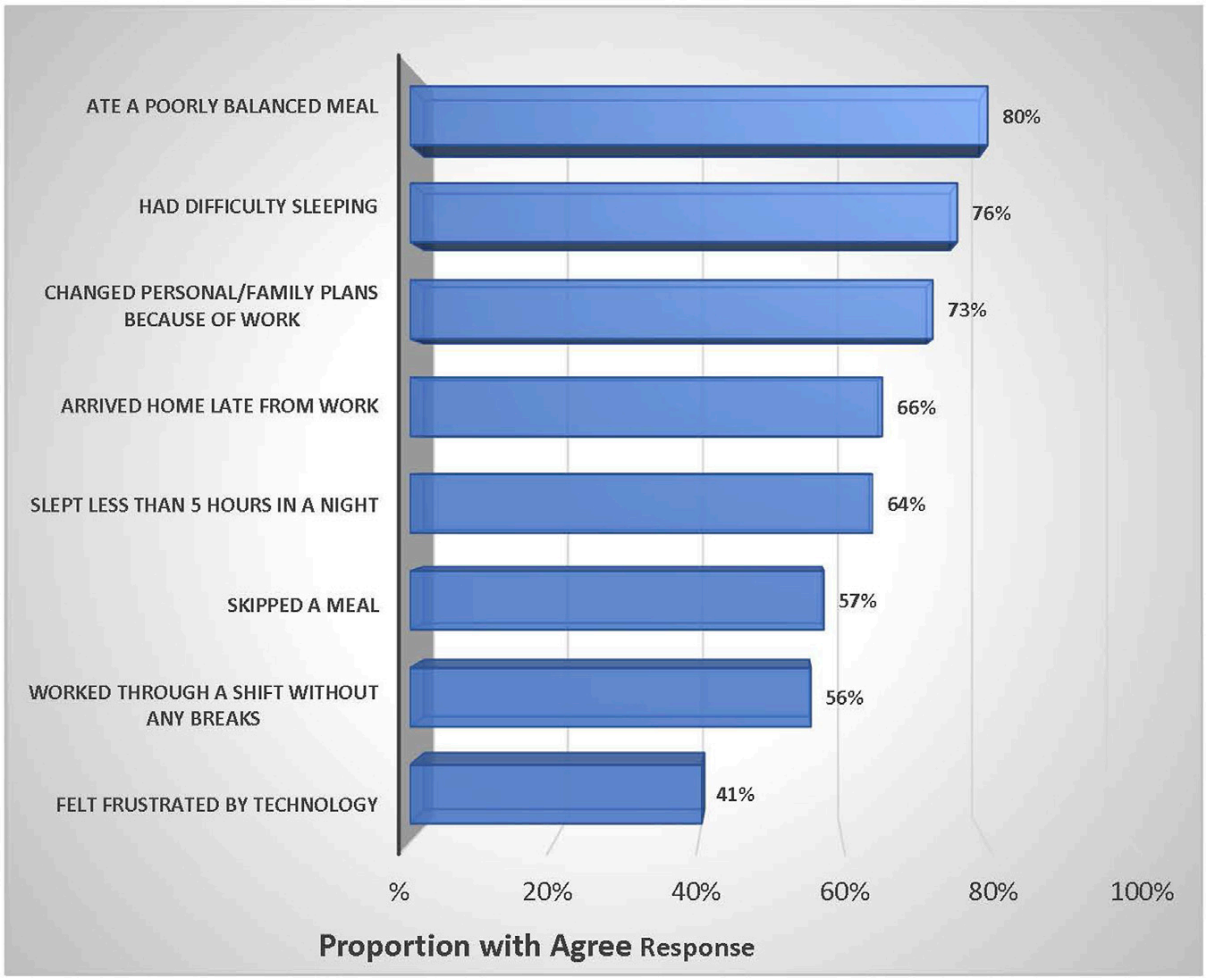
Work–life Balance dimensions among the healthcare professions, (Sudan, 2021).

Figure 1 presents the work-life balance dimensions among the healthcare Professions. Most of the study participants (80%) reported a poorly balanced diet, while three quarters suffered sleep difficulty. Two-thirds (66%) of HCPs arrived home late from work, and 57% skipped a meal during COVID-19. HCPs who worked through a shift without any breaks accounted for over half (56%) of the respondents. Regarding the work-life environment, it was reported that 41% of HCPs felt frustrated by technology.

Table 4 provides The Multiple Linear Regression model for the factors that predict the overall HRQoL scores among the HCPs. The model shows that WLB and (GAD-7) scores significantly predict the level of HRQoL (*p* < 0.001). The results indicate that an increase in the Anxiety scores (GAD-7) by one unit is associated with a decreased HRQoL (β= 0.831, *p* < 0.05). The table further indicated that other healthcare professionals exhibited lower HRQoL scores than the physicians (*p* < 0.05). Moreover, female HCPs also exhibited lower HRQoL scores than the males (*p* < 0.05). Married HCPs were associated with higher levels of HRQoL (*β* = 4.91, *p* < 0.05) compared with single HCPs. Non-smokers were associated with a higher level of HRQoL (*β* = 4.91, *p* < 0.05) than smokers.

**TABLE 4:** Multiple linear regression model for the factors that are associated with the health-related Quality of Life overall scores as a dependent variable, (Sudan, 2021).

Term	Coef	SE Coef	T-Value	*p*-value	VIF	Model summary
Constant	54.70	9.02	6.07	<0.001		F = 13.8 *p*-value= <0.001
Total Anxiety Scores	−0.831	0.131	−6.36	<0.001	1.09	
WLB Scores	−0.969	0.148	6.57	<0.001	1.22	
Age (years)	−0.627	0.337	−1.86	0.063	3.46	
Years of Experience	0.525	0.411	1.28	0.202	3.19	
Gender						
Male	REF					
Female	−5.43	1.61	−3.37	0.001	1.19	
Marital Status						
Single	REF					
Married	4.53	2.19	2.07	0.039	1.23	
Tobacco Use						
Smokers	REF					R^2^ = 0.27
Non-Smokers	4.91	2.44	2.02	0.045	1.16	
Profession						
Physician	REF					
Nurse	4.28	2.72	1.57	0.117	1.16	
Pharmacist	−5.02	3.04	−1.65	0.099	1.13	
Technician	−1.05	3.34	−0.31	0.754	1.08	
Other profession	−5.32	2.36	−2.26	0.025	1.14	
Work place violence						
Yes	REF					
NO	2.98	1.68	1.77	0.078	1.14	

Abbreviation: Coef, *r*egression coefficient; SE Coef, standard error of the coefficient; ref, reference category; REF, reference category.

## Discussion

This study tried to investigate the QoL, mental health, and work-life balance among Sudanese healthcare professionals during the COVID-19 Pandemic. To the best of our knowledge, this is the first national study to assess the experiences of HCPs in Sudan. The COVID-19 Pandemic was unprecedented and affected the healthcare systems in all countries. Healthcare workers were at the frontline of the storm and sacrificed their lives and welfare.

The results revealed moderate to severe anxiety among 35.8% of the study participants. This high level of anxiety among HCPs is consistent with some existing literature [30]. A recent study conducted in Khartoum state in Sudan revealed moderate to severe anxiety in 34.9% of respondents [31]. A wealth of research suggests that excessive work pressure is linked to mental disorders, anxiety, insomnia, distress, and fear of infection [32–34]. A systematic review and meta-analyses revealed that the overall prevalence of stress among HCPs reaches 45% [35]. The mean score of the psychological domain was almost 56, which indicates worse psychological health quality of life. These results indicated that a considerable percentage of HCPs suffered from mental health disorders. This finding is consistent with many published studies during COVID-19 [36–38]. Many factors contribute to the psychological health quality of life, including great pressure at work, life-saving decisions given limited knowledge about the new epidemic, limited resources, and fear of harming family and friends with COVID-19 infection.

The analysis revealed a poor work-life integration in more than half (60.2%) of the HCPs, who expressed dissatisfaction with work-life integration (WLI). This result is consistent with the Khartoum state study [31] and international research [39]. The epidemic created a negative social environment for HCPs including fear, anxiety, and concern for both patients and their loved ones [40]. Some suffered from low social acceptance and high workload that affected their work-family integration [33].

Healthcare professionals experience traumatic events during the COVID-19 pandemic, which make them prone to stress and anxiety. In contrast to many studies [41–43], this research did not document a significant difference in anxiety according to gender, as severe anxiety was documented among 35.1% of males and 36.2% of females. A similar study conducted in Brazil showed that anxiety was more prevalent among females, who were also more likely to develop depression, anxiety, and insomnia than male workers [41].

A large proportion of the study participants reported poor quality of life (33.5%). It is well documented that the healthcare environment is a major public health concern that affected the health of HCPs during COVID-19. A recent review conducted by Kandula and Wake, 2021 concluded that there is a greater impact on the QoL of health professions which is attributed to many issues such as disturbances in physical and mental health [14].

In this study, age and gender were found to be an important factor associated with HRQoL. We found that female HCPs exhibited lower HRQoL scores than their male counterparts. This finding is in line as a study in frontline Brazilian healthcare workers [41] which showed that anxiety was more prevalent among females. The explanation for that according to many systematic reviews reported a higher risk of female HCWs in developing depression, anxiety, and insomnia compared to male workers [5, 14].

One interesting finding was that other healthcare professionals exhibited lower HRQoL scores than the physicians. This finding disagrees with Chalhub, et al (2021), who found that physicians had lower HRQoL. Moreover, the HCPs who experience any type of violence have low HRQoL compared to those who do not [41]. Workplace violence is always associated with dissatisfaction and increased stress [3]. Like most literature, our research revealed the negative association between mental health and HRQoL. Health-related quality of life was worse among HCPs who experienced high stress [17].

Although this research is the first to deal with HCPs quality of life, mental health, and work-life-balance using a relatively representative sample from four states of Sudan, it has some limitations. First, we used a cross-sectional design, so the associations in this research should be interpreted carefully. Second, the self-reported data may affect the accuracy of the study outcomes; however, the level of education in our population and the high response rate may mitigate potential sampling bias. The study did not include important variables, such as direct contact with patients, previous infection of COVID-19, and extra work hours. Further studies are recommended on these issues. Finally, during the final stage of the sampling procedures, the research relied on non-random sampling, which ultimately had an impact on the reliability of the research outcomes.

In conclusion, one-third of the healthcare Professionals in Sudan reported poor Health-related Quality of life-based on the WHO scale during the COVID-19 pandemic. It is also documented that large segments of HCPs have an unequal life-work balance. At the same time, there is a strong association between mental health and HRQol. Health institutions must implement health intervention programs to address mental health concerns among HCPs and promote better-coping strategies in the workplace during pandemics. Further, at a macro level, there is an urgent need for a health policy to address these factors to improve the quality of health of the HCPs and hence improve the standard of patient care in Sudan.

## Abbreviations

HCPs, Healthcare professionals; WLI, work-life integration; WLB, Work-life balance; GAD, General Anxiety Disorder; WHQoL-BREF, Health Organization Quality of Life-BREF; QoL, Quality of Life; HRQoL, Health related Quality of Life.
